# Volumetric brain tumour detection from MRI using visual saliency

**DOI:** 10.1371/journal.pone.0187209

**Published:** 2017-11-02

**Authors:** Somosmita Mitra, Subhashis Banerjee, Yoichi Hayashi

**Affiliations:** 1 Department of Computer Science and Engineering, Institute of Engineering & Management, Kolkata 700091, West Bengal, India; 2 Machine Intelligence Unit, Indian Statistical Institute, Kolkata 700108, West Bengal, India; 3 Department of Computer Science and Engineering, University of Calcutta, Kolkata 700106, West Bengal, India; 4 Dept. of Computer Science, Meiji University, Tama-ku, Kawasaki 214-8571, Japan; University of Pécs Medical School, HUNGARY

## Abstract

Medical image processing has become a major player in the world of automatic tumour region detection and is tantamount to the incipient stages of computer aided design. Saliency detection is a crucial application of medical image processing, and serves in its potential aid to medical practitioners by making the affected area stand out in the foreground from the rest of the background image. The algorithm developed here is a new approach to the detection of saliency in a three dimensional multi channel MR image sequence for the glioblastoma multiforme (a form of malignant brain tumour). First we enhance the three channels, FLAIR (Fluid Attenuated Inversion Recovery), T2 and T1C (contrast enhanced with gadolinium) to generate a pseudo coloured RGB image. This is then converted to the CIE L*a*b* color space. Processing on cubes of sizes *k* = 4, 8, 16, the L*a*b* 3D image is then compressed into volumetric units; each representing the neighbourhood information of the surrounding 64 voxels for k = 4, 512 voxels for k = 8 and 4096 voxels for k = 16, respectively. The spatial distance of these voxels are then compared along the three major axes to generate the novel 3D saliency map of a 3D image, which unambiguously highlights the tumour region. The algorithm operates along the three major axes to maximise the computation efficiency while minimising loss of valuable 3D information. Thus the 3D multichannel MR image saliency detection algorithm is useful in generating a uniform and logistically correct 3D saliency map with pragmatic applicability in Computer Aided Detection (CADe). Assignment of uniform importance to all three axes proves to be an important factor in volumetric processing, which helps in noise reduction and reduces the possibility of compromising essential information. The effectiveness of the algorithm was evaluated over the BRATS MICCAI 2015 dataset having 274 glioma cases, consisting both of high grade and low grade GBM. The results were compared with that of the 2D saliency detection algorithm taken over the entire sequence of brain data. For all comparisons, the Area Under the receiver operator characteristic (ROC) Curve (AUC) has been found to be more than 0.99 ± 0.01 over various tumour types, structures and locations.

## Introduction

The large size of medical image data, the complexity of Features Of Interest (FOIs), and the necessity to process these on time, both accurately and efficiently, are making the job of doctors and radiologists increasingly difficult. Therefore, it has became essential to develop automated delineation of Regions Of Interest (ROIs) and Volumes Of Interest (VOIs) to assist and speedup medical image understanding. Over the last decade cancer has become the deadliest killer worldwide [1]. By the time physical manifestations become evident, often metastasis has set in. This results in failure of local tumor control and poor patient prognosis. Radio-imaging, like magnetic resonance imaging (MRI), computed tomography (CT), positron emission tomography (PET), etc., constitutes one of the best noninvasive approaches for detection, diagnosis, treatment and prognosis of cancer. Particularly, the integration of diverse multimodal information in a quantitative manner provides specific clinical solutions for accurately estimating patient outcome [2].

Among different cancerous tumours of the brain, Glioblastoma multiforme (GBM) remains the most common and lethal form of primary tumor in adults and has poor prognosis. The treatment and diagnosis of GBM is guided by histopathology and imaging findings [3]. Repeated tumor biopsies in the brain is a very challenging problem. Therefore, noninvasive methods like imaging hold immense promise for assessing the state of the tumor. The high spatial resolution of MRI provides minute details of abnormalities, in terms of both shape and volume, in brain tumors. Due to its superior contrast in soft tissue structures, MRIs are routinely used for the diagnosis and characterization of such tumors for disease management. Particularly, MR imaging is very safe becasue it does not involve any exposure to radiation [1].

**Fig 1:**
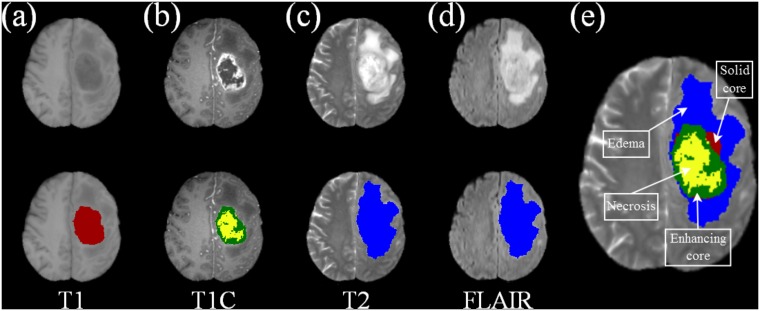
Four primary MR sequences of a 2D GBM slice highlighting different intra-tumoral structures over (a) T1, (b) T1C, (c) T2, and (d) FLAIR, along with (e) composite label map. The bottom row depicts the delineated (a) solid core of the tumor (maroon), (b) enhancing core (green) and necrotic core (yellow), (c-d) whole tumor (blue).

Medical experts manually segment different volumes of interest (VOIs) for detection, diagnosis and prognosis of tumors. Automated medical image analysis, on the other hand, overcomes human bias and can handle large volumes of data. Variation in blood flow (perfusion) within a tumor causes variation in imaging features like necrosis and contrast. Regions of tumor that are poorly perfused on contrast-enhanced *T*1*C*-weighted images may exhibit areas of low (or high) water content on *T*2-weighted images and low (or high) diffusion on diffusion-weighted *FLAIR* (Fluid-Attenuated Inversion Recovery) images [4]. Regions having poor perfusion and high cell density are of particular clinical interest, because they contain cells which are likely to be resistant to therapy. This highlights the utility of superimposing multiple channels of MR imaging, like *FLAIR*, *T*2, and contrast enhanced *T*1*C* components, in identifying and extracting heterogeneous tumor region(s) [Fig 1] [5].

Humans can easily identify the salient (or relevant) parts of an image mainly due to the attention mechanism of the human visual system. “Visual saliency”, coined by Ullman and Sha’ashua [6] was extended by Itti *et al*. [7] towards the development of a computational architecture. Computational models of saliency take images as input and generate a topographical map of how salient or attention grabbing each area of the image can be to a human observer. Such models seem to predict based on certain aspects of human eye movement [8].

Visual saliency can be defined as the outcome of comparing a region with its surrounding, with respect to unpredictability, contrast and rarity [9, 10]. Saliency detection methods can be broadly classified into (i) biological [7, 11], (ii) fully computational [12, 13] and (iii) hybrid [14, 15]. Algorithms employing the bottom-up strategy detect saliency by using low-level features, like color, intensity, orientation. Those using the top-down strategy include some learning from the training data involving the position or shape of a salient object. It has been observed that often attention is immediately drawn to a salient item, in spite of the existence of many other items (or distracters), without any need to scan the entire image. A visually salient region is typically rare in an image, and contains highly discriminating information. This concept is, therefore, expected to have a major bearing towards the fast identification of tumor from a medical image.

Computer-Aided Detection (CADe) can be of help to doctors and radiologists in identifying abnormalities, which are comparatively rare, in a medical image. The objective of this research is to improve the present CADe systems by minimizing user interactions, thereby saving precious time of doctors while reducing possibility of human error. Application of visual saliency to medical images is being studied in literature. Jampani *et al*. [16] investigated the usefulness of three popular computational saliency models, extended from the natural scene framework, to detect abnormalities in chest X-ray and color retinal images. Visual saliency was also applied for automated lesions detection [17, 18] from retinal images. Alpert *et al*. [19] developed a medical saliency model for detecting lesions and microcalcifications in mammograms, MRIs of brain, and stenoses of angiographic images. However sufficient validation study, with respect to ground truth, was not provided. Erihov *et al*. [20] designed a shape asymmetry-based saliency model for detection of tumors from brain MRI and breast mammograms.

Banerjee *et al*. [5] designed an algorithm, based on the concept of visual saliency, for localizing and segmenting 2D GBM tumor regions from multi-sequence MRI. A region was considered as visually salient (or attention grabbing) depending on its rarity in the image, thereby signifying the content of some discriminating information (like abnormalities, in the context of medical imaging). Multi-sequence MR images were integrated to generate pseudo-colored MRI for efficiently detecting the whole tumor region in 2D. A bottom-up saliency detection strategy, incorporating spatial distance between image patches, was used to highlight the salient region(s) in the image. It was established [5] that the performance was better than related state-of-the-art 2D methodologies in medical imaging. However the applicability of this model *BA* being constrained to 2D MR images, one fails to generate accurate and uniform volumetric saliency for 3D MR images.

### Contribution

Multi-level MR images constitute vast 3D data, with each voxel representing distinct physical measurements of a tissue-dependent characteristic. In the present era of precision medicine, where even slight differences in disease manifestation are seen as potential areas for new intervention strategies, one cannot afford to forego any part of this valuable information. Therefore precise volumetric analysis assumes prime importance; so that accuracy of subsequent segmentation of VOI, extraction of radiomic features, and finally decision-making, get the least compromised.

**Fig 2:**
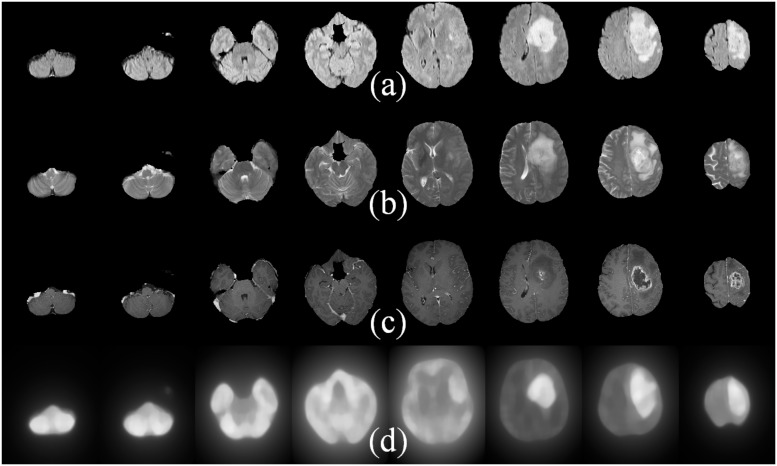
Saliency maps for High-grade glioma samples. MR slice numbers: 13, 19, 35, 46, 67, 81, 100, 121 (left to right) by (a) FLAIR, (b) T2, and (c) T1C sequences. (d) Saliency maps generated in 2D [5].

Although an extension of *BA* [5] to 3D may be envisaged by applying the algorithm over a stack of 2D MR slices, its utility becomes doubtful—particularly, in the case of those slices towards the outer portion of the VOI. It is because the foreground (ROI) in such slices, encompassing the three MR sequences, may be too small as compared to the background region; with the algorithm generating false positive results. This is evident from the MR images constituting the starting and ending slices of the 3D stack of brain images, as depicted by the leftmost and rightmost slices (columns) in Fig 2(a)–2(d). As the saliency is computed based on the mean color difference between patches, the algorithm erroneously highlights the entire small foreground region in such 2D slices as tumour [Fig 2(d)].

In order to circumvent such problem of losing valuable information, we propose the novel 3D volumetric approach to saliency detection *PR*. Working with a single plane, as in the case of 2D saliency computation, and then juxtaposing them can result in an uneven outline of the tumour region—due to insufficient utilization of available information. Therefore, by the terminology 3D we intend better usage of available data along all three basic dimensions, viz. length, breadth and height, corresponding to the major axes. This helps in making a precise volumetric analysis and generates accurate incorporation of the MR channel information.

The saliency of a region in an image can be defined by how starkly different it is from its surrounding area. When a 3D image is decomposed to a series of 2D slices, we observe that employing a single dimensionality of comparison results in the top and bottom portions of the brain (in slices at either end) to be standing out as salient. This is because, being a small portion of the image, it stands out from the rest of the dark background. On the contrary, if we were to apply a tri (*xy*, *yz*, *xz*) plane comparison on the same 3D image then even if the beginning or ending slices occupied a smaller area of the brain on (say) the *xy* plane, yet the *yz* and *xz* planes would impart a weighted contribution towards restoring the resultant saliency component of the image.

**Fig 3:**
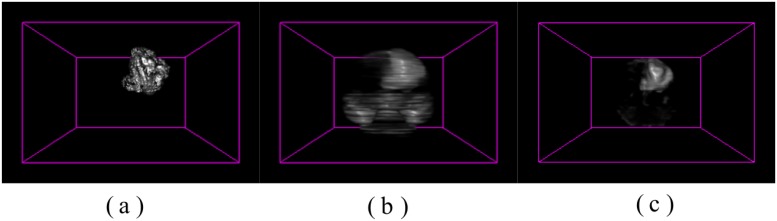
3D saliency maps. (a) Ground truth, with saliency map generated (b) using Ref. [5] and (c) the proposed method *PR*.

The proposed algorithm *PR* thus effectively minimizes any incorrect classification of regions, and prevents erroneous highlighting of small foreground regions. This is illustrated in Fig 3. Moreover, in order to maximise the accuracy of the salient region, each MR sequence fed into its respective channel is normalized by its maximum value such that associated hidden features are made prominent and the boundaries get clarified. Then it is converted into a channel of bit depth 8, thereby catering to 256 possible thresholds of singular intensity. This helps in maintaining a common ground for comparison and analysis, while catering to the discernibility between various regions of the tumour along each MR channel by minimizing misclassification during saliency detection.

The remainder of the article is organized as follows. We describe the data used and the methodology, while outlining the contribution of the proposed volumetric algorithm for tumour detection using visual saliency. This is followed by a presentation and analysis of the experimental results, both qualitatively and quantitatively, to illustrate the improvement of the 3D algorithm with reference to its earlier 2D version.

## Materials and methods

### Ethics statement

“Brain tumor image data used in this work were obtained from the MICCAI 2015 Challenge on Multimodal Brain Tumor Segmentation (http://www.imm.dtu.dk/projects/BRATS2015) organized by B. Menze, A. Jakab, S. Bauer, M. Reyes, M. Prastawa, and K. Van Leemput. The challenge database contains fully anonymized images from the following institutions: ETH Zurich, University of Bern, University of Debrecen, and University of Utah. All human subjects data was publicly available de-identified data. Therefore, no Institutional Review Board approval was required” [21, 22].

### Data used

Brain tumor image data used in this work was obtained from the MICCAI 2015 Challenge on Multimodal Brain Tumor Segmentation organized by B. Menze, A. Jakab, S. Bauer, M. Reyes, M. Prastawa, and K. Van Leemput. There are 274 glioma cases, of which 220 are of High-grade (HG) and 54 of Low-grade (LG), with four MR sequences T1, T1C, T2, and FLAIR being available for each patient. The challenge database contains fully anonymized images collected from ETH Zurich, University of Bern, University of Debrecen, and University of Utah. All images are linearly co-registered and skull stripped. Since all data on human subjects is publicly available and de-identified, therefore no Institutional Review Board approval was required. Utilizing the three MRI sequences, viz. FLAIR, T1C and T2, the individual portions of the glioma have been highlighted to generate a 3D saliency map. It preserves the innate characteristics of the region in each sequence, while accentuating the entirety of the area affected.

### Pseudo-colouring

Colour images in the digital spectrum usually encompass three colour channels of red, green and blue (RGB). Each of these channels can be broken down to individually represent gray scale images of 16 bits each, and subsequently recombined in 3! ways to reproduce the original colour image. Utilizing an analogous methodology, we generate a semblance of a colour image (pseudo coloured) using the three gray scale images from sequences *FLAIR*, *T*1*C* and *T*2 [5]. This engenders a 48 bit RGB colour image, containing more information than any of the three channels individually.

### Methodology

Considering the three RGB channels in order, FLAIR MRI sequence was loaded into the R channel, the T1C sequence was mapped into the G channel and T2 into the B channel. The resulting 48 bit colour image is utilised for all future processing.

One of the major bottlenecks of this approach is the resultant pixel values, which are individually difficult for the machine to interpret and display. This hampers the prominence of the end result and berates the validity of the algorithm. Saliency with respect to brain tumours being essentially described by how prominently the glioma region can stand out from the rest of the brain matter, it requires human comprehension. Without the distinctions being discernible to the human eye, it is impossible to assess the pragmatism of this algorithm; especially as an aid to medical practitioners. Therefore we enhance each channel by uniformly distributing the 8 bits of each singular RGB channel to the spectrum of intensities upto the existential maximum intensity value. This increases the understandability, and generates a more visually apparent and highly accentuated saliency map.

In order to maximise the accuracy of the salient region, each MRI sequence is normalized to enhance hidden characteristics and boundaries. Then the processed image is converted to its corresponding L*a*b* format, which is useful in increasing the perceivability of the various colour components associated. It helps enhance the luminosity and chromaticity of an image with respect to each other as given in Ref. [5].

Another issue which cropped up during the development of this procedure was the volume-wise voxel saliency generation. While it is relatively easier to generate the saliency map for a 2D image, the complexity increases in the 3D space. This is because in 3D the computation of the degree of saliency depends not only on the neighbourhood voxels of the same plane, but also on those of all other intersecting planes. Hence those slices containing insignificant portions of the tumor may adversely affect the degree of saliency of the VOI. Moreover brain MRI sequences are not always perfect, with many images in the database having unwarranted intrusion of light along one or more axes [21].

To overcome this difficulty we used all three major planes, viz. *xy*, *yz*, and *xz* planes, to calculate the average saliency. Using a cubic scaling factor *k* = 4, 8, and 16, each 3D image space was broken down into cubes of its respective sizes. For example, size 4 cubes had the information of its 64 neighbours imbibed into one single voxel. Thereby we could successfully achieve our objective using only the three major axes, with the neighbourhood information getting extracted from the voxel intensity.

At the same time it is important to generate a uniform breaking down mechanism to serve as a viable decomposition tool for all images. Since the 3D images in the sourced database are of varied sizes, we transmute it into a dimensional scope of *M* × *M* × *N*, where *M* and *N* are divisible by *k* (*k* = 4, 8, 16). Thereby, the entire 3D image volume is broken down into three separate groups representing volumes of size 4 (64 pixels), 8 (512 pixels) and 16 (4096 pixels) respectively. Each non-overlapping cube is represented by its mean L*a*b* values, thus decomposing it into a single unit with the values from its surrounding pixels stored as its integral property. The number of cubes correspond to the number of pixels in the saliency map, which would be of dimension (*M*/*k* × *M*/*k* × *N*/*k*) with each cube being of size (*k* × *k* × *k*). Therefore, the *i*th cube of the image *I*(*V*_*i*_), 1 ≤ *i* ≤ (*M*/*k* × *M*/*k* × *N*/*k*), can be represented by its mean L*a*b* value as

$$\begin{array}{c} V_{i,j,l}^{\bar{L}{*}^{\;}}=\frac{\sum I(V_{i,j,l}^{L^{*}})}{k\times k\times k},V_{i,j,l}^{\bar{a}{*}^{\;}}=\frac{\sum I(V_{i,j,l}^{a^{*}})}{k\times k\times k},V_{i,j,l}^{\bar{b}{*}^{\;}}¯=\frac{\sum I(V_{i,j,l}^{b^{*}})}{k\times k\times k}. \end{array}$$ (1)

The ability to effectively discern the glioma from the rest of the brain matter is an essential parameter to be considered in this algorithm. The salient features of the image are ascribed to the superimposition of the three MRI channels which accentuate the region of the tumour. Therefore the saliency of each cube is compared with the variegation of colours with respect to all other cubes along the major planes (*xy*, *yz*, *xz*) of the image. Utilizing the simplest of all approaches, the basic difference between the mean L*a*b* colour values of each such cube pair is represented using the Euclidean norm. This allows mapping the pseudo-colored cube pairs, graded on a normalized scale of 0–255 shades, to enhance the colour difference over each individual channel. Generating a basis for colour difference also helps in finding a common ground for perpetuating and representing any visually apparent change in the composition of each cube.

**Fig 4:**
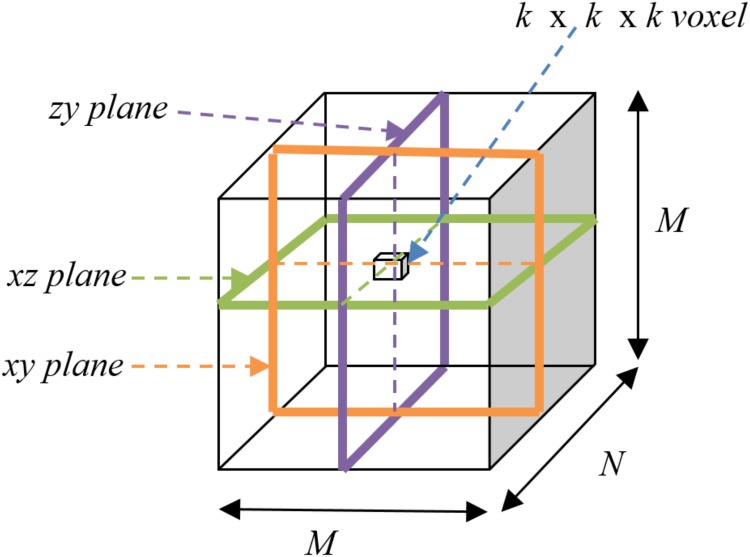
Schematic illustrating voxel-level computation in algorithm *PR*.

Consider Fig 4. Since the evaluation of saliency *S*_*c*_ for each *k* × *k* × *k* voxel *V*_*i*,*j*,*l*_ is performed only along either of the three major axes (at a time), while excluding *V*_*i*,*j*,*l*_ itself during distance computation, therefore we formulate the expression as

$$\begin{array}{ll} S_{c}\left(V_{i,j,l}\right)=\sum_{\left(i=x\vee j=y\vee l=z\right)\wedge \neg \left(i=x\wedge j=y\wedge l=z\right)} & \sqrt{{\left(V_{i,j,l}^{\bar{L}*}-V_{x,y,z}^{\bar{L}*}\right)}^{2}+{\left(V_{i,j,l}^{\bar{a}*}-V_{x,y,z}^{\bar{a}*}\right)}^{2}+{\left(V_{i,j,l}^{\bar{b}*}-V_{x,y,z}^{\bar{b}*}\right)}^{2}}\\ & \forall \left(i,j,x,y\right)\in \left\{1,\ldots ,M/k\right\},\forall \left(l,z\right)\in \left\{1,\ldots ,N/k\right\}. \end{array}$$ (2)

Thereafter, the colour variation of a cube with that of the rest of the cubes along the major axes of the image are summed. If the value of this sum is sizeable then it is considered to be a salient cube.

It is often observed that the salient regions are congregated over adjacent spaces, with the non-salient regions being present anywhere in the image. Thus we can surmise that the probability of a region being salient is higher if it is nearer to a pre-established salient cube; while those that are located farther away have a lower probability of being salient. Based on this assumption we incorporate the distance of the cube from the other cubes under consideration, along with their difference in color, to reformulate the volumetric saliency expression as

$$\begin{array}{ccc} S(V_{i,j,l}) & = & \sum_{\left(i=x\vee j=y\vee l=z)\wedge \neg (i=x\wedge j=y\wedge l=z\right)}\frac{1}{1+d(V_{i,j,l},V_{x,y,z})}\times S_{c}\left(V_{i,j,l}\right). \end{array}$$ (3)

Since neighboring regions of a salient one are also likely to be salient, therefore a lower *d*(*V*_*i*,*j*,*l*_,*V*_*x*,*y*,*z*_) contributes towards a higher effective value of *S*(*V*_*i*,*j*,*l*_).

When an observer views a far-off scene, the focus lies on the entire salient region(s). Again when the same scene is viewed at a closer range, the observer tends to pay greater attention to the details within the salient region. This property of the human visual attention mechanism was adopted in 2D through the evaluation of saliency maps at multiple scales [5].

Extending this concept to the 3D scenario in our volumetric algorithm *PR* by considering volume sizes *k* × *k* × *k*, *k* = 4, 8, 16. We were able to clearly highlight the salient object, at a higher resolution, by partitioning the image into smaller sized volumes. A volumetric saliency map can be considered to be a probability map, with the intensity of a voxel indicating its chance of belonging to the tumor region in the original image. Although the saliency map for a larger patch can help in accurately locating a salient object, its resultant blurring causes disappearance of most details.

Re-scaling is performed to bring back the saliency maps to the original image size (*M* × *M* × *N*) using Bilinear interpolation [23]. Let ${\hat{S}}_{k}$ denote the interpolated image at its original size, as generated from the saliency map *S*^*k*^ at scale *k*. Since the properties of a region depend on the voxels within it, saliency prediction is governed by both its size and scale. Thus our algorithm is simultaneously employed over multiple scales for capturing the salient region(s) in the MR image at different levels of resolution. Those region(s) consistently highlighted over different resolutions are deemed to be the ones most likely to be salient. Therefore we superimpose these saliency maps, corresponding to the different scales, for computing the final map

$$\begin{array}{c} S=\sum_{k=4,8,16}r^{k}\times {\hat{S}}^{k}, \end{array}$$ (4)

where $r^{k}=\frac{1}{3}$ is the weight of the saliency map ${\hat{S}}_{k}$. This helps in detecting the tumour region.

### Algorithm *PR*

Assuming the 3D image to be of dimension *M* × *M* × *N*, the rubric of the proposed volumetric algorithm is explicated as follows.

1. Initialize FLAIR sequence to R channel, T1C sequence to G channel and T2 sequence to B channel.
2. Normalize each channel by its maximum intensity pixel, to highlight regions and clarify boundaries, followed by scaling to uniform bit depth.
3. Convert the RGB channel to its L*a*b* counterpart.
4. **Repeat Steps 5-8** for dimension size *k* = 4, 8, 16
5. Generate a map of dimension *M*/*k* × *M*/*k* × *N*/*k*
6. Calculate the mean of *k* × *k* × *k* pixels and store it as the voxel value for *i*, *i* ∈ *M*/*k* × *M*/*k* × *N*/*k*, using Eq (1).
7. Compute the saliency *S*(*V*_*i*_), ∀*i* ∈ *M*/*k* × *M*/*k* × *N*/*k*, by Eqs (2) and (3) along the three major axes.
8. Combine the saliency map at resolution *k*, with 1/3 weight, to generate the final volumetric saliency map *S* by Eq (4).
9. Apply median filter on the final saliency map for smoothening. This helps the algorithm focus on the core region of the VOI in the resized image.
10. Display the final saliency map.

## Results

The performance of the proposed volumetric saliency-based algorithm *PR*, incorporated directly at the voxel level, was compared to that of its 2D predecessor *BA* [5] (working at pixel level, and extended to the 3D domain for comparison). The 2D saliency model *BA* needed to be applied to individual 2D slices of an MRI volume, with their juxtaposition resulting in a simulated 3D saliency map. Results are evaluated in terms of the match with the ground truth involving the whole tumor region, encompassing the intra-tumoral structures, namely “edema”, “nonenhancing (solid) core”, “necrotic (or fluid-filled) core”, and “non-enhancing core”. Tumour structure definitions often have certain ambiguities, especially when clear boundaries are hard to define. As a method of standardization all subjects, annotated by several experts, have the results subsequently fused to obtain a single consensus segmentation for each subject. The MICCAI BRATS 2015 database, used for the evaluation of the algorithm, utilizes this as the ground truth for effectiveness detection in our algorithm.

A saliency map is portrayed as a gray scale image of similar dimensions as that of the original image, where the intensity of a pixel signifies its significance for belonging to the glioma region in the original image. While an intensity 0 (pure black) implies least importance, an intensity of 255 (pure white) corresponds to being of utmost significance. We thresholded the 3D saliency maps in the range of 0–255 to generate binary masks, which are then evaluated against different metrics of comparison.

Precision refers to the percentage of correctly classified salient voxels over the whole image, whereas recall corresponds to the portion of voxels from the ground truth which get detected correctly. Although recall and precision are inversely proportional to each other, we cannot do without either. Therefore we have maximized both of these. The entire range of gray levels in the image is explored, for exhaustive thresholding, in order to generate two classes; with the positive class representing the VOI and the negative class being treated as the background. Area Under the Curve (AUC) is estimated by analyzing the Receiver Operator Characteristic (ROC) from these thresholded images. While the true positive rate (TPR) is the proportion of saliency values at actual locations above a threshold, the proportion of voxels corresponding to the non-tumor regions of the ground truth (but wrongly classified as tumor regions) contribute towards the false positive rate (FPR).

**Fig 5:**
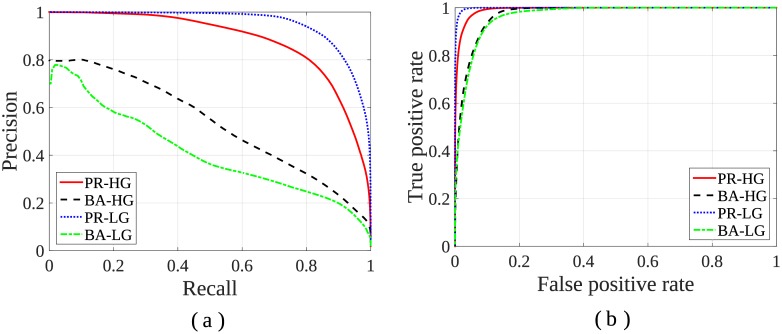
Comparative study over varying thresholds [0-255] on saliency map for HG and LG tumour images. (a) Precision-Recall, and (b) ROC curves.

The quantitative performance of our 3D algorithm *PR* is evaluated by computing the precision and recall, along with the TPR and FPR over these thresholded saliency maps. The precision-recall and ROC curves are plotted in Fig 5 by averaging over the set of images from each of the two data groups (HG, LG). The corresponding Area Under Curve (AUC) values for ROC in case of algorithm *PR* are 0.992 ± 0.01 (HG) and 0.998 ± 0.01 (LG). The values are indicative of a close match to the manual detection. On the other hand, for algorithm *BA* the AUC values are 0.96 ± 0.1 (HG) and 0.95 ± 0.2 (LG). Therefore, it is evident that our 3D algorithm *PR* generates statistically better precision-recall and ROC curves as compared to that of the earlier 2D saliency approach *BA*, in both groups.

**Fig 6:**
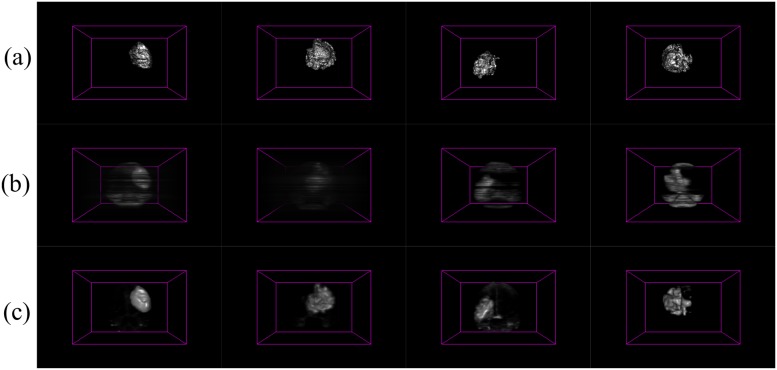
3D saliency maps for four HG glioma patients. (a) Ground truth, with 3D saliency map generated by (b) *BA* [5], and (c) algorithm *PR*.

**Fig 7:**
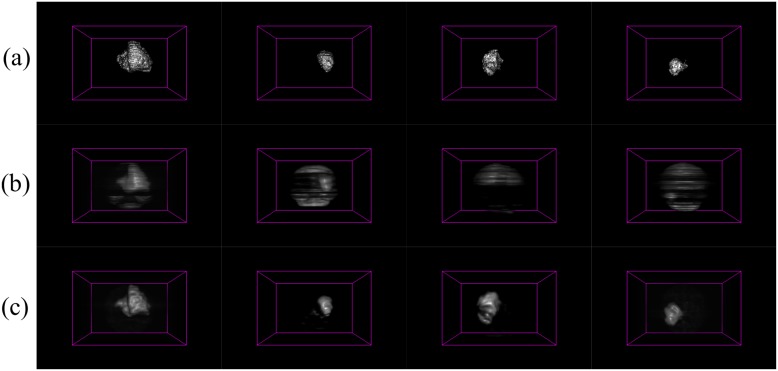
3D saliency maps for four LG glioma patients. (a) Ground truth, with 3D saliency map generated by (b) *BA* [5], and (c) algorithm *PR*.

The axial, sagittal and coronal views of the brain are used to represent the saliency mapped cube generated as a result of our algorithm *PR*. When we compare this to the result obtained by *BA*, we notice a few distinct differences. As discussed earlier, the main utility of *PR* lies in accurately modeling the tumour in the uppermost and lowermost regions of the MR image. This is clearly evident in case of both HG and LG brain images, from the inaccuracy observable from the volumetric saliency maps of Figs 6(b) and 7(b) by *BA* [5], as compared to the corresponding ground truth in Figs 6(a) and 7(a).

### Discussion

In our proposed algorithm *PR*, instead of considering each slice of the 3D image as a 2D component, we broke down the entire image region into ordered cubes of uniform dimensions. From this it becomes apparent that for a cubic image dimensionality of *M* × *M* × *N* = 240 × 240 × 240, we would have a 13,82,400 pixel arrangement. Reducing this to the cubic classification spectrum, for dimension side *k* = 4, there are *M*/*k* × *M*/*k* × *N*/*k* = 60 × 60 × 60 pixels in the image; which is equivalent to *n* = 2,16,000 non-overlapping cubes. Analogously for dimension side *k* = 8, there are 30 × 30 × 30 pixels in the 3D image arrangement which is equal to *n* = 27,000 non-overlapping cubic partitions. To calculate the relative mean L*a*b* values for each individual cubic representation would require (*n*−1) comparisons each, and therefore *n* × (*n*−1) total comparisons, where *n* indicates the number of averaged voxels. Hence for dimension side *k* = 4, with number of cubes being 2,16,000, the calculation of the relative mean L*a*b* would require n*(*n*−1) = 2,16,000*2,15,999 = 466557,84,000 comparisons. Similarly for side dimension *k* = 8, there is the need for 27,000*26,999 = 7289,73,000 comparisons. This results in a higher time complexity and makes the algorithm very slow.

On the other hand, if we were to look at the initial criterion for *k* = 4, where each 4 × 4 × 4 voxel was used to calculate the mean L*a*b* value, then we observe that the neighbourhood values are already present in the averaged voxel. Thus comparison along all pixels of the decomposed 3D image becomes unnecessary.

In algorithm *PR* we use only the three major axes for a 3D co-ordinate system, as seen in Fig 4.

Therefore for a cube side dimension of *k* = 4, the cubic non-overlapping arrangement has dimension *M*/*k* × *M*/*k* × *M*/*k* = 60 × 60 × 60 with the number of comparisons getting reduced to 60 × 60 along each axis; resulting in a total of 2,16,000 × 3 × 60 × 60 (# averaged voxels of interest × # axes × area dimension along each axis), which is 23328,00,000 comparisons by Eq (2). We can observe how the number of comparisons has reduced from 466557,84,000 to 23328,00,000, which is 1/20 times the original value. On repeating the same with cube side dimension *k* = 8, we get 27,000 × 3 × 30 × 30 or 729,00,000, which is approximately 1/10 times the value of 7289,73,000. Thus there is reduction in comparison time, as a measure of improved computational complexity. The result produced using the three major axes is sufficient to generate coherent tumour regions, and prevents damage from distortions produced by external light sources entering the source image or from partially derived source images. This is because here equal weightage is not provided to the extremities of the image slices, where distortions typically occur.

With the advent of modern day technology the MR images assume varied resolutions, with each having a different bit/pixel depth. Previously MR channel images had the pixel depth set to 8, while now some machines use 16. To establish a common ground and a uniform comparison basis, we had to scale down each image to its 8 bit form. While working with MR images, it has been observed that sometimes the minute details get lost due to smaller difference in intensity values alongside each pixel. To overcome this problem, we scale each individual channel to its 8 bit counterpart and at the same time normalize using the maximum intensity value of the image. Thereby the highest intensity value is treated as 1, with a range of intensities from 0 to 1. Scaling it to an 8 bit form involves multiplication by 255, resulting in a 0-255 threshold image channel. This helps in developing the final image where each point is highlighted and appropriately attenuated to correspond to each region of the glioma. Such pre-processing of the image helps with the correct classification of the final result, and generates a more accurate and uniform output form.

## Conclusion

Computer-Aided Detection (CADe) focuses on an automated and fast detection, localization and segmentation of the VOI. Here we developed a novel volumetric saliency-based algorithm for the efficient detection of GBM tumor(s) from multi-channel MRI. The formation of a 3D view representing the saliency of the tumour region in the entire brain is useful to generate a proper outline of the tumour area. When the boundaries are well defined then the malignancy of the tumour can be properly estimated by the medical practitioners. This will help in upholding the integrity of the term CADe, as the entirety of the tumour region can be detected.

While saliency detection in the 2D version of the algorithm (*BA*) was used to estimate only on a single cross sectional view of the tumour, the new 3D volumetric saliency detection algorithm (*PR*) enabled a more complete detection of the existing tumour(s) as estimated over the 3D image of the human brain. This contribution is of utmost significance due to its pragmatic usability and potential applicability in real time saliency detection for patients suffering from GBM. Use of the 3D approach is shown to elicit more accurate VOIs (as compared to the ground truth), both on qualitative and quantitative terms, by minimizing loss of valuable information with respect to the 3D extrapolation of the previous 2D version [5]. This is expected to lead to improved segmentation and extraction of radiomic features, for subsequent decision-making.

## References

[1] Mitra S, Uma Shankar B. Medical image analysis for cancer management in natural computing framework. Information Sciences. 2015;306:111–131. doi: 10.1016/j.ins.2015.02.015

[2] Gatenby RA, Grove O, Gillies RJ. Quantitative Imaging in Cancer Evolution and Ecology. Radiology. 2013;269:8–14. doi: 10.1148/radiol.13122697 24062559 10.1148/radiol.13122697 PMC3781355

[3] Holland EC. Glioblastoma multiforme: The terminator. Proceedings of the National Academy of Sciences. 2000;97:6242–6244. doi: 10.1073/pnas.97.12.6242 10.1073/pnas.97.12.6242 PMC33993 10841526

[4] Zinn PO, Sathyan P, Mahajan B, Bruyere J, Hegi M, Majumder S, et al A novel Volume-Age-KPS (VAK) Glioblastoma classification identifies a prognostic cognate microRNA-gene signature. PLOS ONE. 2012;7:e41522 doi: 10.1371/journal.pone.0041522 22870228 10.1371/journal.pone.0041522 PMC3411674

[5] Banerjee S, Mitra S, Uma Shankar B, Hayashi Y. A Novel GBM Saliency Detection Model Using Multi-Channel MRI. PLOS ONE. 2016;11(1):1–16. doi: 10.1371/journal.pone.0146388 10.1371/journal.pone.0146388 PMC4709039 26752735

[6] Ullman S, Sha’ashua A. Structural saliency: The detection of globally salient structures using a locally connected network. In: Proceedings of the Second International Conference on Computer Vision; 1988. p. 321–327.

[7] Itti L, Koch C, Niebur E. A model of saliency-based visual attention for rapid scene analysis. IEEE Transactions on Pattern Analysis and Machine Intelligence. 1998;20:1254–1259. doi: 10.1109/34.730558

[8] Foulsham T, Underwood G. What can saliency models predict about eye movements? Spatial and sequential aspects of fixations during encoding and recognition. Journal of Vision. 2008;8:1–17. doi: 10.1167/8.2.6 18318632 10.1167/8.2.6

[9] Achanta R, Estrada F, Wils P, Süsstrunk S. Salient region detection and segmentation In: Computer Vision Systems. Springer; 2008 p. 66–75.

[10] Ma YF, Zhang HJ. Contrast-based image attention analysis by using fuzzy growing. In: Proceedings of the Eleventh ACM International Conference on Multimedia. ACM; 2003. p. 374–381.

[11] Walther D, Koch C. Modeling attention to salient proto-objects. Neural Networks. 2006;19:1395–1407. doi: 10.1016/j.neunet.2006.10.001 17098563 10.1016/j.neunet.2006.10.001

[12] Rosin PL. A simple method for detecting salient regions. Pattern Recognition. 2009;42:2363–2371. doi: 10.1016/j.patcog.2009.04.021

[13] Zhang L, Tong MH, Marks TK, Shan H, Cottrell GW. SUN: A Bayesian framework for saliency using natural statistics. Journal of Vision. 2008;8:32 doi: 10.1167/8.7.32 19146264 10.1167/8.7.32 PMC7360059

[14] Bian P, Zhang L. Biological plausibility of spectral domain approach for spatiotemporal visual saliency In: Advances in Neuro-Information Processing. Springer; 2009 p. 251–258.

[15] Harel J, Koch C, Perona P. Graph-based visual saliency In: Advances in Neural Information Processing Systems; 2006 p. 545–552.

[16] Jampani V, Sivaswamy J, Vaidya V, et al. Assessment of computational visual attention models on medical images. In: Proceedings of the Eighth Indian Conference on Computer Vision, Graphics and Image Processing. ACM; 2012. p. 80.

[17] Deepak KS, Chakravarty A, Sivaswamy J, *et al*. Visual saliency based bright lesion detection and discrimination in retinal images. In: 10th IEEE International Symposium on Biomedical Imaging. IEEE; 2013. p. 1436–1439.

[18] Quellec G, Russell SR, Abramoff MD. Optimal filter framework for automated, instantaneous detection of lesions in retinal images. IEEE Transactions on Medical Imaging. 2011;30:523–533. doi: 10.1109/TMI.2010.2089383 21292586 10.1109/TMI.2010.2089383 PMC13677053

[19] Alpert S, Kisilev P. Unsupervised detection of abnormalities in medical images using salient features In: SPIE Medical Imaging. International Society for Optics and Photonics; 2014 p. 903416–903421.

[20] Erihov M, Alpert S, Kisilev P, Hashoul S. A Cross Saliency Approach to Asymmetry-Based Tumor Detection In: Medical Image Computing and Computer-Assisted Intervention â€“ MICCAI 2015. vol. 9351 of Lecture Notes in Computer Science. Springer International Publishing; 2015 p. 636–643.

[21] Menze B, et al The Multimodal Brain Tumor Image Segmentation Benchmark (BRATS). IEEE Transactions on Medical Imaging. 2014;34:1993–2024. doi: 10.1109/TMI.2014.2377694 25494501 10.1109/TMI.2014.2377694 PMC4833122

[22] Kistler M, Bonaretti S, Pfahrer M, Niklaus R, Büchler P. The Virtual Skeleton Database: An Open Access Repository for Biomedical Research and Collaboration. Journal of Medical Internet Research. 2013;15(11):e245 doi: 10.2196/jmir.2930 24220210 10.2196/jmir.2930 PMC3841349

[23] Erdem E, Erdem A. Visual saliency estimation by nonlinearly integrating features using region covariances. Journal of Vision. 2013;13:11 doi: 10.1167/13.4.11 23509407 10.1167/13.4.11

